# Gustatory dysfunction in relation to circumvallate papilla’s taste buds structure upon unilateral maxillary molar extraction in Wistar rats: an *in vivo* study

**DOI:** 10.12688/f1000research.19684.1

**Published:** 2019-09-20

**Authors:** Sana Mostafa, Heba M. Hakam, Amal El-motayam

**Affiliations:** 1 Department of Oral Biology, Faculty of Dentistry, Cairo University, Cairo, Egypt

**Keywords:** Molars extraction, taste sensation, circumvallate papilla, taste buds

## Abstract

**Background**: The interaction between taste sensation and dentoalveolar innervation is still under research.  teeth loss can alter taste thresholds in humans, but the underlying mechanisms are still obscure. This study investigated the effect of unilateral maxillary molars extraction on the structure of circumvallate papilla in rats.

**Methods**: Thirty-two male Wister rats, aged 3-4 months were randomly distributed into four groups (one control and 3 experimental ) each including 8 animals. The rats were euthanized 3, 6 or 9 weeks following the procedure. The changes in trough length and the taste buds structure and number of both sides of CVP were investigated using routine histological examination followed by statistical analysis.

**Results**: the trough toward the extraction side was obviously shorter with a noticeable decrease of taste buds’ number than the non-extraction side. Taste buds were reduced in size and most of them showed signs of degeneration which was more evident in group II followed by group III, less deformity detected in group IV in comparison to the preceding 2 experimental groups. the non-extraction side of all experimental groups showed normal trough length and generally normal histology of taste buds.

**Conclusions**: Maxillary molars extraction has a degenerative effect on the structure of  taste buds and gustatory epithelium which were more marked at the extraction side and showed improvement upon elongation of follow up period

## Introduction

Taste is an essential sensation that is mediated via taste buds (TBs) which are specialized neuroepithelial structures distributed predominantly over the dorsal surface of the tongue ^1– 3^, and undergo continuous renewal throughout adult life ^4, 5^.

The circumvallate papilla (CVP) is a major compartment of the gustatory system in the tongue that harbors more than 500 taste bud openings in its trough ^6, 7^. This gathering of taste organs in one specified place, make it easier to detect different changes under study.

 Moreover, the impact of nerve injury on fungiform taste buds appears to be less severe than on circumvallate taste buds, thus making it a good candidate to study neural variations ^8, 9^.

The gustatory system is unique in that taste buds innervation is essential to taste bud cell turnover ^10^. In the rat tongue, the single central CVP is innervated by the glossopharyngeal nerve bilaterally ^7, 11^.

Taste sensation is modulated by different factors including the integration of gustatory information with other sensory modalities such as olfaction, food texture, temperature and pain (hot spices) through the trigeminal nerve ^12^. Other factors include nerve supply and mechanical stimuli that are affected by the integrity of masticatory apparatus ^13^.

Taste signals synapse to the nucleus of the solitary tract (NST) located in the medulla ^14^. An interaction between dental and gustatory afferent neurons in the NST has been previously discussed ^9, 15– 17^. In addition, teeth loss can alter taste thresholds in humans, but the underlying mechanisms are still obscure ^15, 18, 19^.

This interaction has clinical implications as occlusion impairment by teeth extraction (dental deafferentation) can be considered a possible cause of taste alteration ^15^.

It was reported that after unilateral transection of glossopharyngeal nerve in rat, the TBs loss in CVP was 12% rather than a 50% which indicate bilateral innervation of the remaining taste buds ^20^. However, another study suggested that all the TBs in the rat CVP are bilaterally innervated except the upper two thirds of the outer trough walls which are innervated by ipsilateral glossopharyngeal nerve ^21^.

The purpose of this study was to compare between both sides of the CVP regarding trough length and histopathological changes in the TBs structure and number secondary to unilateral maxillary molars extraction and to identify if unilateral extraction will cause damage to glossopharyngeal nerve on the same side of CVP only or may extend to the other side.

Rats were the animal of choice for this experiment because of their similarity to human anatomy regarding turnover time of taste buds, and innervation pattern of the tongue ^12, 13^.

## Methods

### Animals

A total of 32 male Wistar rats weighing 150–200 g, aged 3–4 months were used in this study. The animals were obtained from and housed in the Animal House, Faculty of Medicine, Cairo University. The animals were maintained in an air conditioned animal house under controlled room temperature 25±2°C with 12/12 h light/dark cycle and were allowed unlimited access to powdered soft food and water. Rats were housed in (standard polycarbonate cages (Pretty industries, Model:CR5). Control rats were placed as 4 animals per cage, while the experimental animals were placed one per cage until wounds had fully recovered in order to control infection.

All procedures were refined to reduce the negative impact on animals including administration of anesthesia prior to the procedure and gentle handling of the animal during extraction to prevent any tissue laceration or damage. After the experiment the animals received antibiotics to prevent infection, and analgesics to manage any pain or discomfort. The cages were cleaned frequently, they were supplied with wood chips substrate, colored tubes and ropes to ensure enriched environment for the animal.

The welfare of the animals was assessed prior to, during and after the experimental period by the attending veterinarian who intervened in case of pain or distress signs according to the ethical protocols for animal treatment that were supervised by the animal facilities, Faculty of Medicine, Cairo University. The study was approved by the Institutional Animal Care and Use Committee, Cairo University (Approval no. CUIIIS1016).

### Sample size calculation

Sample size calculation based on previous research by Hsu *et al*., 2014; that reported the difference in the trough depth between the control and experimental (28 days) groups was 28 ±13 µm. Using power 90% and 5% significance level, 6 rats in each group was calculated to be sufficient to be able to reject the null hypothesis that the population means of the experimental and control groups are equal. That number was increased to 8 rats in each group to compensate for possible loss of animals during breeding. Sample size calculation were performed using PS: Power and Sample Size Calculation software Version 3.1.2 (Vanderbilt University, Nashville, Tennessee, USA).

### Animal grouping and study design

 Rats were randomly distributed by Random Sequence Generator program ( randomizer.org) into four groups (one control and 3 experimental) each including 8 animals. Implementation of the allocation was done as follow: numbers from 1 to 32 were written on folded papers that were placed in opaque sealed envelopes, matching of the rats with the numbers were done blindly through the technician in charge at the animal house, each rat was attached to its number till the end, then the numbers were opened and the rats were allocated in their groups according to the program recommendations.

 The control (group I) were anesthetized without tooth extraction (sham operation) and euthanized after 3 weeks. In the experimental groups, all maxillary molars of the right side were extracted (early in the morning) under general anesthesia by an intraperitoneal injection of ketamine/xylazine (ketamine 40–100 mg/kg IP/ xylazine 5–13 mg/kg IP, Trittau, Germany) for 60–80 minutes ^22^. Extraction were performed using Halsted Curved Mosquito Hemostatic Forceps, Germany 15921-G with the tongue retracted by a tweezer. Postoperatively, antibiotics were administrated (cefotaxime10mg/Kg IV, Eipico, Egypt) for 3 days ^23^. Analgesics were administrated (Ketoprofen (Anafen ^®^, Merial) 5mg/kg I.M) ^24^ once daily for up to 3 days.

Animals were euthanized with an intra-venous overdose of sodium thiopental (80mg/kg) ^25^ (Anapental 1,P2-55-011, Sigma Tec Pharma –Egypt) (as a humane method that cause rapid euthanasia with minimal discomfort to the animals) after extraction at 3 weeks (group II), 6 weeks (group III) and 9 weeks (group IV) ( Table 1).

**Table 1.** Animal grouping and study design.

Group	Number of animals	Intervention (Extraction)	Animal Euthanasia
**Group I**	8	No	3 weeks
**Group II**	8	Yes	3 weeks
**Group III**	8	Yes	6 weeks
**Group IV**	8	Yes	9 weeks

The first euthanization date for the current study (3 weeks) were assigned according to **Huang & Lu, 1996** study as they found that taste buds of the CVP were diminished 3 weeks after neurectomy ^26^. The other intervals (6 and 9 weeks) were set in order to investigate the probability of recovery from any potential damage. The CVP was dissected out according to the animal’s tongue anatomy. The CVP is situated most posteriorly at the center of the base of the tongue, the site of the CVP in the dissected rat tongue was identified, then the tissue specimen was isolated by a horizontal cut anterior to the papilla and two oblique cuts posteriorly. Tissues were fixed in 10% calcium formol (No:8012-95-1, El-Gomhouria co., Egypt).

 Serial 5-microns thick sections were prepared using LEICA RM2255 microtome and picked up on glass slides (No: 7101, Yancheng Jingwei Chemicals Co., Ltd-china) for histological haematoxylin and eosin staining (No:L16220, L13960, El-Gomhouria co., Egypt).

The sections were deparaffinized and rehydrated by immersing them successively for ~5 min with agitation in xylene, 100% ethanol, and 70% ethanol. Then the slides were rinsed in running tap water at room temperature for at least 2 min. The sections were stained in hematoxylin solution for 3 min then placed under running tap water for 1 min. The sections were stained in 1% eosin Y solution for 2 min. The sections were dehydrated with two consecutive immersions in 95% alcohol, then two immersions in 100% alcohol for 30 sec each. The alcohol was then extracted with two consecutive immersions in xylene ^27^. Blinding was applied for the primary animal care giver and the assessor of the histological and statistical results.

The specimens were examined using Leica DM300 light microscopic (Leica Microsystems, Inc., Switzerland).

### Histomorphometric analysis

**Figure 1.**
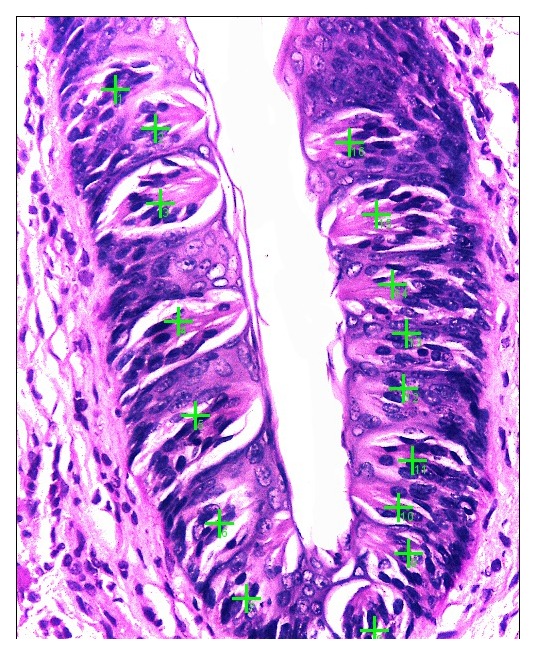
Photomicrograph demonstrating the method of taste buds counting.

TBs’ number for each specimen were counted using an objective lens of magnification (x40) for all groups by light microscope. TBs were marked by green crosses and counted by Image J Version:1.52p computer system ( Figure 1).

**Figure 2.**
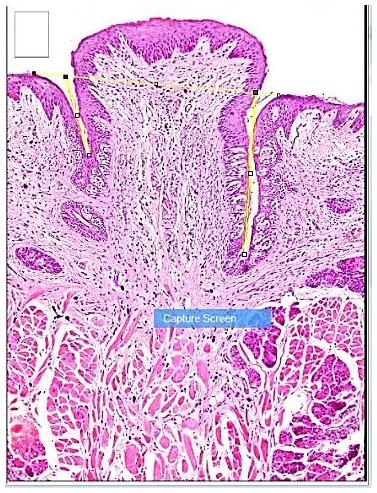
Photomicrograph showing method of trough length measurement. The trough opening was determined by a solid line drawn between the 2 troughs, the trough depth was marked by two lines, from top to bottom.

The length of both trough sides for each specimen was measured using an objective lens of magnification (x10) for all groups using light microscope. The length was measured using computer system ( Figure 2).

### Statistical analysis

Comparisons between groups were performed using one-way analysis of variance (ANOVA). This was followed by Tukey’s post hoc test for pairwise comparisons when ANOVA revealed a significant difference. Independent t test was used to compare both trough sides using IBM SPSS 18.0 version 21 for windows (SPSS Inc., Chicago, IL, USA). The significance level was set at p ≤ 0.05.

## Results

### Histological results

**Figure 3.**
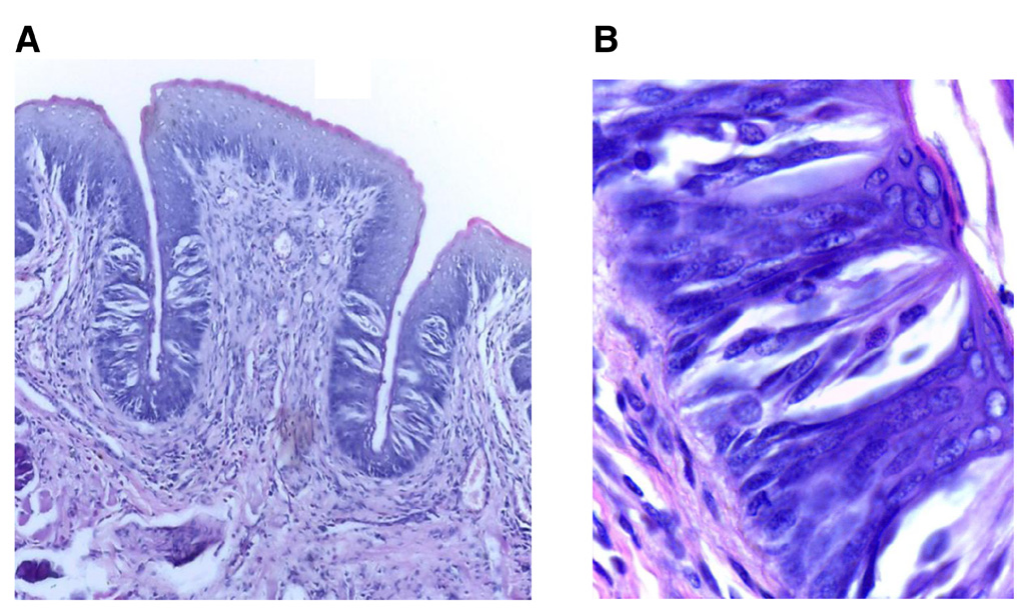
( **A**) Photomicrograph of control group (І) showing normal circumvallate papilla surrounded by a deep narrow trough of nearly equal length on both sides and gustatory epithelium with normal taste buds (H&E, orig. Mag.X100). ( **B**) Higher magnification showing mature barrel shaped taste buds with taste pores, different taste cells types with prominent nuclei including supporting cells, gustatory receptor cells and basal cells (H&E, orig. Mag.X1000). H&E - haematoxylin and eosin, Mag - magnification.

**Figure 4.**
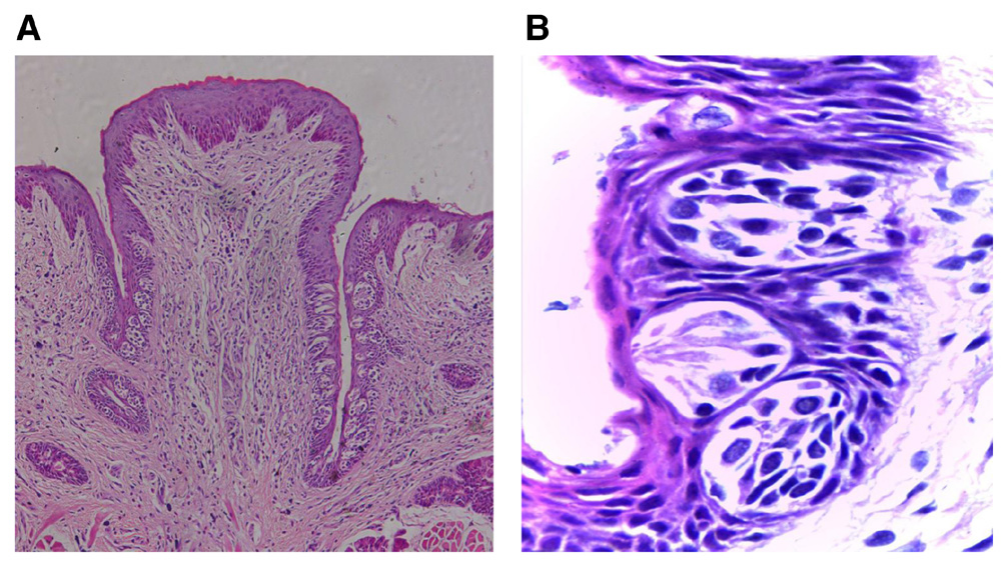
( **A**) Photomicrograph of group (ІI) showing deformity of the papilla general outline with shorter troughs toward the extraction side with fewer taste buds, and normal trough length toward the non-extraction side containing increased taste buds number (H&E, orig. Mag.X100). ( **B**) Higher magnification showing immature taste buds toward the extraction side arranged at different levels and decreased in size with signs of degeneration (H&E, orig. Mag.X1000). H&E - haematoxylin and eosin, Mag - magnification.

**Figure 5.**
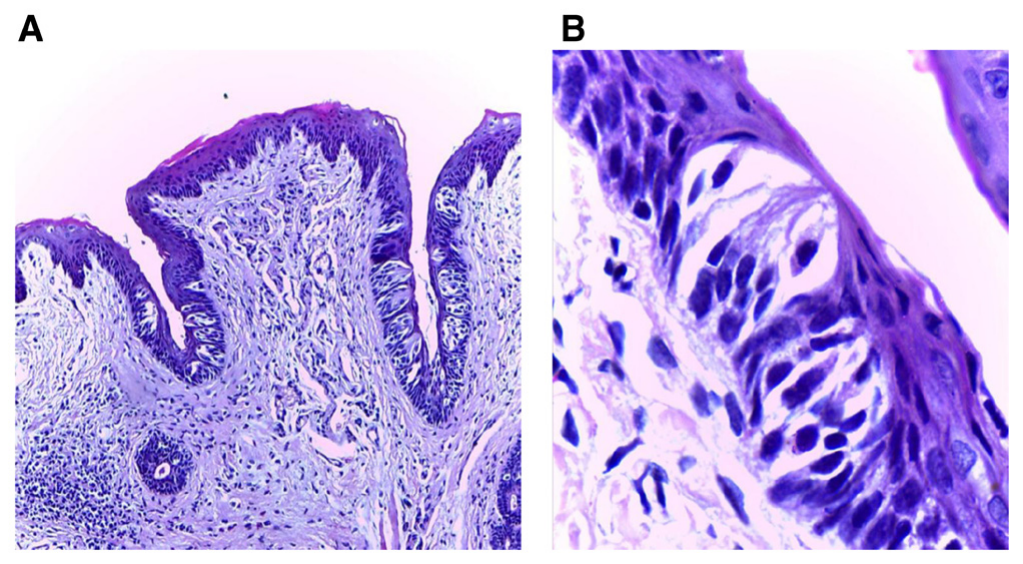
( **A**) Photomicrograph of group (ІII) showing shorter trough length toward the extraction side with decreased number of taste buds, focal area of inflammatory cells infiltration beneath the shorter trough and normal taste buds number in the other trough (H&E, orig. Mag.X100). ( **B**) Higher magnification showing extraction side with decreased taste bud number with darkly stained nuclei (H&E, orig. Mag.X1000). H&E - haematoxylin and eosin, Mag - magnification.

**Figure 6.**
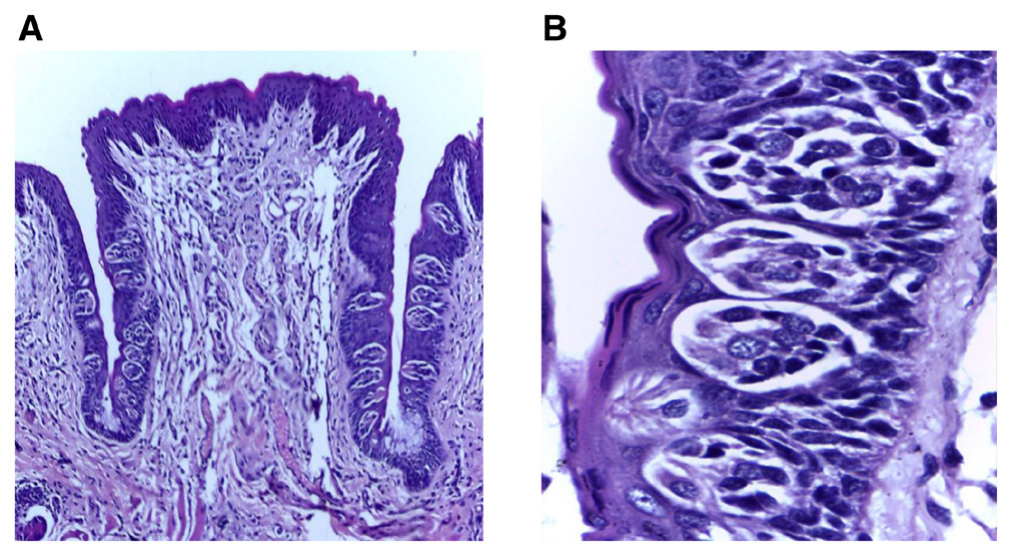
( **A**) Photomicrograph of group (ІV) showing trough toward the extraction side was nearly the same length as the other side, with increased number of taste buds in comparison to previous experimental groups (H&E, orig. Mag.X100). ( **B**): Higher magnification of extraction side showing small size-buds with a few darkly stained nuclei (H&E, orig. Mag.X1000). H&E - haematoxylin and eosin, Mag - magnification.

**Figure 7.**
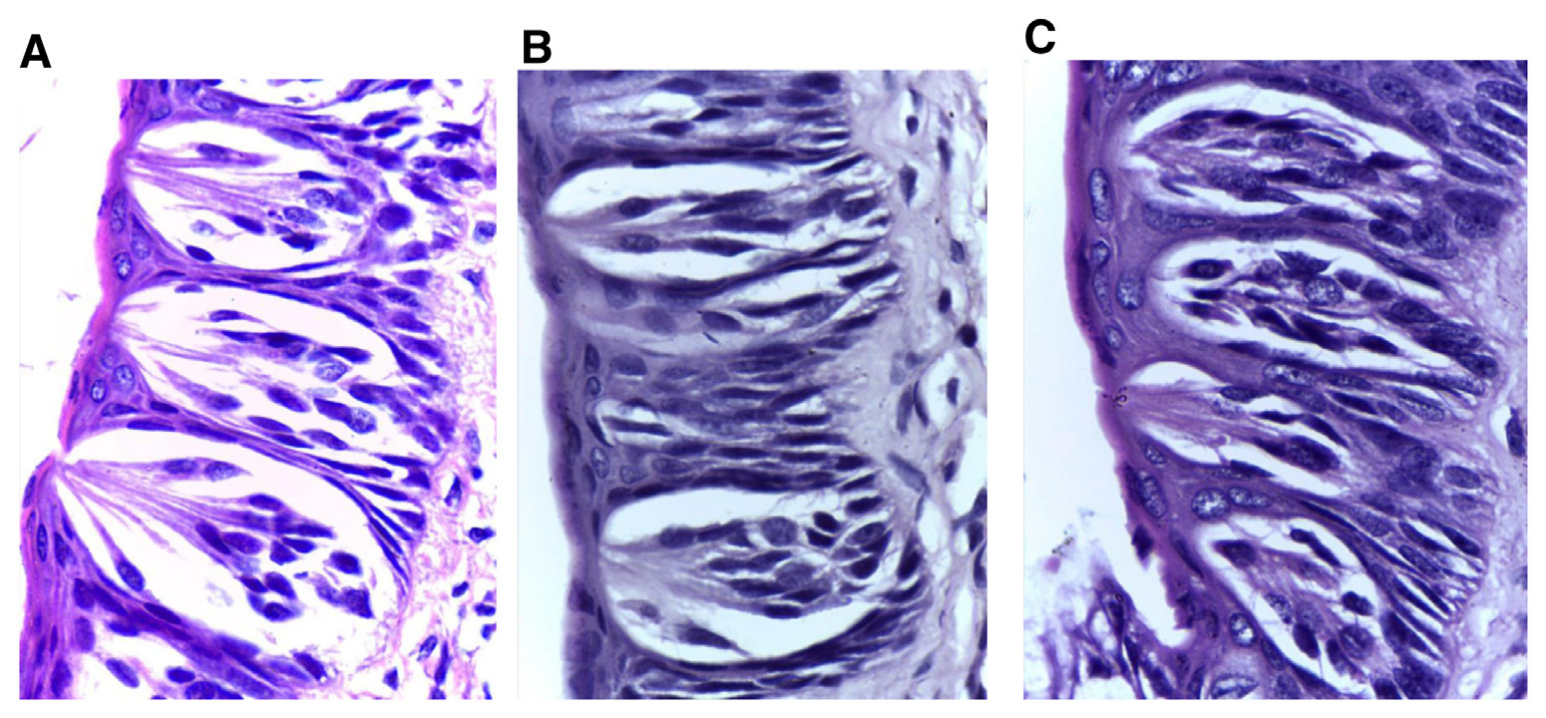
Taste buds of the non-extraction side in all experimental groups ( **A**) group (II), ( **B**) group (III) and ( **C**) group (IV) showing generally normal histology except for slight enlargement (in groups II & III) and a few darkly stained nuclei (H&E, orig. Mag.X1000). H&E - haematoxylin and eosin, Mag - magnification.

Analysis of the control group samples revealed normal CVP surrounded by even trough lengths with normal TB histology and number ( Figure 3). In groups II and III, the papillae showed slight deformity of the general outline as the trough toward the extraction side was obviously shorter with noticeable decrease of TBs’ number than the non-extraction side. TBs were reduced in size and most of them showed signs of degeneration which was most evident in group II, followed by group III ( Figure 4, Figure 5). A similar situation was observed in group IV with less deformity of the general outline, as the right side revealed an increase in length with apparent increase in TB number in comparison to the preceding two experimental groups. TBs exhibited nearly normal shape, with some degenerated taste cells ( Figure 6). TBs of the non-extraction side in all experimental groups showed generally normal histology except for slight enlargement and few darkly stained nuclei ( Figure 7). Raw microscope images are available as underlying data ^28– 31^.

### Statistical results

**Figure 8.**
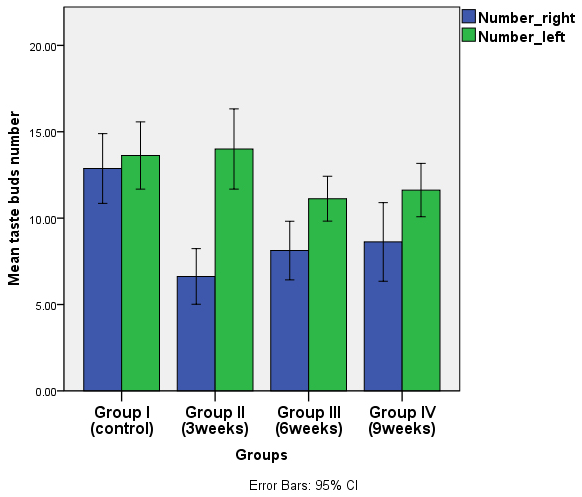
Bar chart showing mean number of taste buds for both sides of troughs in different groups. (blue columns for the extraction side and green columns for non-extraction side).

**Figure 9.**
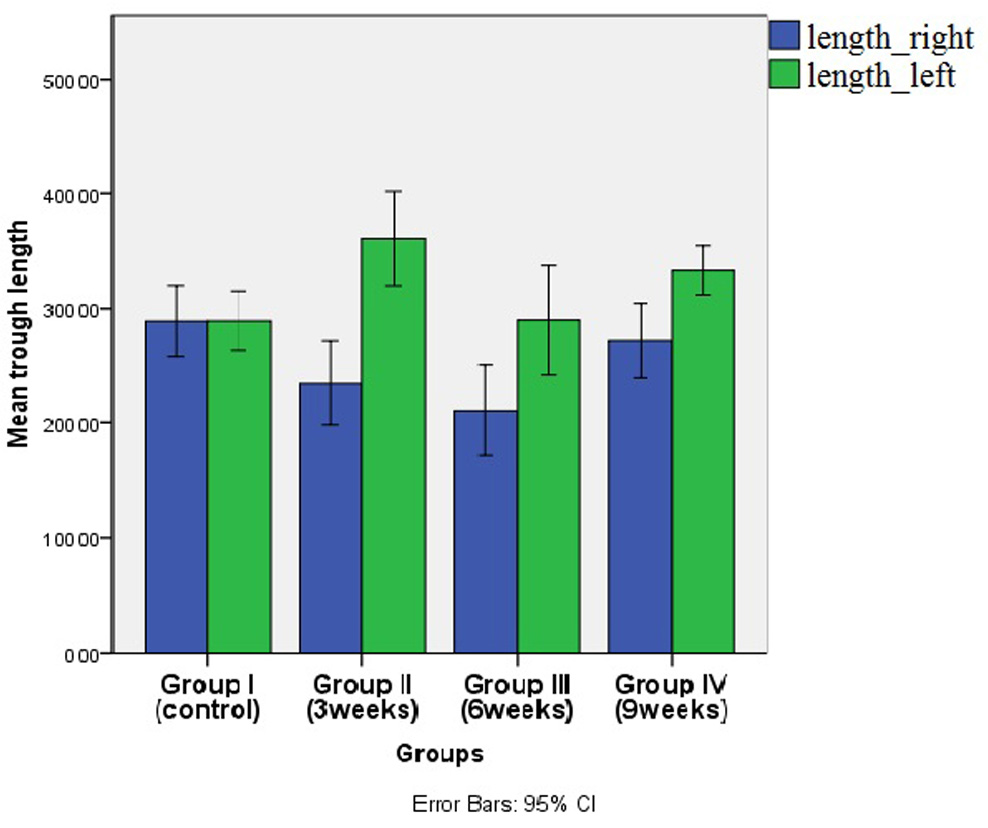
Bar chart showing mean trough length (µm) in the both sides in different groups. (blue columns for the extraction side and green columns for non-extraction side).

TB number and trough length variation between the two sides (extraction & non-extraction) were recorded among all experimental groups. It was noticed that non-extraction side recorded a higher mean number and length within each group, while the highest mean value was recorded in group II. This difference was not statistically significant in the controls, but was significant in groups II, III & IV (p ≤ 0.05). ( Table 2 and Table 3, Figure 8 and Figure 9, underlying data ^32^).

**Table 2.** Comparison of taste bud number between right and left side within the same group (independent t test).

		Mean	SD	Mean difference	Std error of difference	t	P
**Group I(Control)**	Right	12.88	2.42	-0.750	1.186	-0.632	0.537 NS
	Left	13.63	2.33				
**Group II(3weeks)**	Right	6.63	1.92	-7.38	1.19	-6.175	0.00 *
	Left	14.00	2.78				
**Group III(6weeks)**	Right	8.13	2.03	-3.00	0.90	-3.319	0.005 *
	Left	11.13	1.55				
**Group IV(9weeks)**	Right	8.63	2.72	-3.00	1.16	-2.579	0.024 *
	Left	11.63	1.85				

Significance level p<0.05, *significant, NS=non significant

**Table 3.** Comparison of length of troughs (µm) between right and left side within the same group (independent t test).

		Mean	SD	Mean difference	Std error of difference	t	P
**Group I(Control)**	Right	289.17	36.75	-0.322	16.916	-0.019	0.985NS
	Left	289.49	30.64				
**Group II(3weeks)**	Right	235.09	44.34	-125.65	23.38	-5.375	0.00 *
	Left	360.73	49.05				
**Group III(6weeks)**	Right	211.49	47.71	-78.72	26.21	-3.003	0.01 *
	Left	290.20	56.75				
**Group IV(9weeks)**	Right	272.29	38.47	-61.01	16.34	-3.735	0.002 *
	Left	333.30	25.60				

Significance level p<0.05, *significant

## Discussion

Taste is a highly dynamic and complex process. The interaction between gustation and somatosensation of the tongue is still under investigation ^13^. The consequences of dental deafferentation as one of the possible causes of taste disorders, is unclear and therefore an area for further investigation.

The current results are in accordance with **Hsu *et al.*, 2014** who observed decreased trough lengths with fewer TB numbers of the CVP 28 days following bilateral maxillary molars extraction in rats ^13^. They suggested that this might be due to peripheral or central neuroplastic changes induced by dental deafferentation. Similarly, **Braud *et al.*, 2012** uncovered the interaction between some of dental afferents from the trigeminal nerve & gustatory neurons in the NST ^16^.

The decreased TBs number toward the extraction side in the current study was in agreement with **Huang & Lu, 1996** who studied unilateral glossopharyngeal nerve neurectomy, and concluded that the taste buds on the same side of the CVP decreased significantly in number during the first two weeks, and disappeared completely by week three ^26^. Moreover, **Ichimori *et al.*, 2009** reported that denervation of gustatory epithelium causes apoptosis in all types of taste buds, resulting in rapid degeneration ^33^.

On the other hand, **Guth 1963** found that following unilateral glossopharyngeal nerve transection in the CVP of rats, the mean loss of taste buds in the outer trench walls was greater on the operated than the unoperated sides. However, they found that this difference was not statistically significant ^20^. **Guagliardo & Hill, 2007** demonstrated that TBs of fungiform papilla on the uncut side increased in size ^34^. This might explain enlarged TBs on the non-extraction side.

The highest mean values were recorded in group II towards the non-extraction side, which could be explained as a compensatory mechanism for the damage that has occurred at the other side.

Improved results observed in group IV regarding trough length and TBs’ number are probably due to the recovery of the gustatory epithelium with time. This is in agreement with **Lim & Green, 2008** who postulated that nerve damage in certain region would augment remaining taste signals in the neighboring area ^35^.

Study limitations may include that the process of counting TBs, which needs to be more precise, as the same taste bud may appear in different sections, like counting the total number of taste buds per papilla by scoring only TBs of maximum dimensions. A more accurate investigation methods could be used in further studies rather than mere TBs counting, like using reverse transcription polymerase chain reaction (RT-PCR) for detection of the expression of certain genes.

The current study has clinical implications, as it identified a correlation between normal occlusion and taste sensation, and it could be considered as an aid in explaining the etiology of taste loss. According to the current study results, it is important to screen subjects for dental status when planning taste studies, especially in sensory analyses. Subjects with recent multiple extractions should not be included in such studies as taste sensation is likely to have been affected. These findings also could help to distinguish the effect of normal aging from disease-related changes in taste perception.

The current study provides a step forward in predicting the effect of molar extraction and the impact of unilateral glossopharyngeal damage on taste sensation in humans. However, further investigations and observational studies are needed to prove that connection.

## Conclusions

Maxillary molars extraction has a degenerative effect on the structure of taste buds and the gustatory epithelium which were more marked at the extraction side, and showed improvement upon elongation of follow up period.

## Data availability

### Underlying data

Harvard Dataverse: Dataset1. Control group (group I). https://doi.org/10.7910/DVN/UZ9OL0 ^28^

This project contains the following underlying data:

- c1.tif (Microscope image, 10x magnification of control sample 1)
- c1a.tif (Microscope image, 40x magnification of control sample 1)
- c1b.tif (Microscope image, 40x magnification of control sample 1)
- c2.tif (Microscope image, 10x magnification of control sample 2)
- c2a.tif (Microscope image, 40x magnification of control sample 2)
- c2b.tif (Microscope image, 40x magnification of control sample 2)
- c3.tif (Microscope image, 10x magnification of control sample 3)
- c3a.tif (Microscope image, 40x magnification of control sample 3)
- c3b.tif (Microscope image, 40x magnification of control sample 3)
- c4.tif (Microscope image, 10x magnification of control sample 4)
- c4a.tif (Microscope image, 40x magnification of control sample 4)
- c4b.tif (Microscope image, 40x magnification of control sample 4)
- c5.tif (Microscope image, 10x magnification of control sample 5)
- c5a.tif (Microscope image, 40x magnification of control sample 5)
- c5b.tif (Microscope image, 40x magnification of control sample 5)
- c6.tif (Microscope image, 10x magnification of control sample 6)
- c6a.tif (Microscope image, 40x magnification of control sample 6)
- c6b.tif (Microscope image, 40x magnification of control sample 6)
- c7.tif (Microscope image, 10x magnification of control sample 7)
- c7a.tif (Microscope image, 40x magnification of control sample 7)
- c7b.tif (Microscope image, 40x magnification of control sample 7)
- c8.tif (Microscope image, 10x magnification of control sample 8)
- c8a.tif (Microscope image, 40x magnification of control sample 8)
- c8b.tif (Microscope image, 40x magnification of control sample 8)
- control x1000a.tif (Microscope image, 100x magnification of control sample)
- control x1000b.tif (Microscope image, 100x magnification of control sample)

Harvard Dataverse: Dataset2. Group II samples. https://doi.org/10.7910/DVN/FOFV89 ^30^

This project contains the following underlying data:

- g2 (100x) ex..tif (Microscope image, 100x magnification of group II sample of extraction side)
- g2 (100x) ex.a.tif (Microscope image, 100x magnification of group II sample of extraction side)
- g2 (100x) ex.b.tif (Microscope image, 100x magnification of group II sample of extraction side)
- g2 (100x) non ex.a.tif (Microscope image, 100x magnification of group II sample of extraction side)
- g2 (100x) non ex.b.tif (Microscope image, 100x magnification of group II sample of extraction side)
- g2-1(10x).tif (Microscope image, 10x magnification of group II sample 1)
- g2-1(40x) ex..tif (Microscope image, 40x magnification of group II sample 1 of extraction side)
- g2-1(40x) non ex..tif (Microscope image, 40x magnification of group II sample 1 of non-extraction side)
- g2-2(10x).tif (Microscope image, 10x magnification of group II sample 2)
- g2-2(40x) ex..tif (Microscope image, 40x magnification of group II sample 2 of extraction side)
- g2-2(40x) non ex..tif (Microscope image, 40x magnification of group II sample 2 of non-extraction side)
- g2-3(10x).tif (Microscope image, 10x magnification of group II sample 3)
- g2-3(40x) ex..tif (Microscope image, 40x magnification of group II sample 3 of extraction side)
- g2-3(40x) non ex..tif (Microscope image, 40x magnification of group II sample 3 of non-extraction side)
- g2-4(10x).tif (Microscope image, 10x magnification of group II sample 4)
- g2-4(40x) ex..tif (Microscope image, 40x magnification of group II sample 4 of extraction side)
- g2-4(40x) ex..tif (Microscope image, 40x magnification of group II sample 4 of extraction side)
- g2-5(10x).tif (Microscope image, 10x magnification of group II sample 5)
- g2-5(40x) ex..tif (Microscope image, 40x magnification of group II sample 5 of extraction side)
- g2-5(40x) non ex..tif (Microscope image, 40x magnification of group II sample 5 of non-extraction side)
- g2-6(10x).tif (Microscope image, 10x magnification of group II sample 6)
- g2-6(40x) ex..tif (Microscope image, 40x magnification of group II sample 6 of extraction side)
- g2-6(40x) non ex..tif (Microscope image, 40x magnification of group II sample 6 of non-extraction side)
- g2-7(10x).tif (Microscope image, 10x magnification of group II sample 7)
- g2-7(40x) ex..tif (Microscope image, 40x magnification of group II sample 7 of extraction side)
- g2-7(40x) non ex..tif (Microscope image, 40x magnification of group II sample 7 of non-extraction side)
- g2-8(10x).tif (Microscope image, 10x magnification of group II sample 8)
- g2-8(40x) ex..tif (Microscope image, 40x magnification of group II sample 8 of extraction side)
- g2-8(40x) non ex..tif (Microscope image, 40x magnification of group II sample 8 of non-extraction side)

Harvard Dataverse: Dataset3. Group III samples. https://doi.org/10.7910/DVN/KYNFDS ^30^

This project contains the following underlying data:

- g3 (100x) ex.1.tif (Microscope image, 100x magnification of group III sample of extraction side)
- g3 (100x) ex.2.tif (Microscope image, 100x magnification of group III sample of extraction side)
- g3 (100x) ex.3.tif (Microscope image, 100x magnification of group III sample of extraction side)
- g3 (100x) non ex.1.tif (Microscope image, 100x magnification of group III sample of non-extraction side)
- g3 (100x) nonex.2.tif (Microscope image, 100x magnification of group III sample of non-extraction side)
- g3-1 (10x).tif (Microscope image, 10x magnification of group III sample 1)
- g3-1(40x) ex..tif (Microscope image, 40x magnification of group III sample 1 of extraction side)
- g3-1(40x) non ex..tif (Microscope image, 40x magnification of group III sample 1 of non-extraction side)
- g3-2 (10x).tif (Microscope image, 10x magnification of group III sample 2)
- g3-2(40x) ex..tif (Microscope image, 40x magnification of group III sample 2 of extraction side)
- g3-2(40x) non ex..tif (Microscope image, 40x magnification of group III sample 2 of non-extraction side)
- g3-3 (10x).tif (Microscope image, 10x magnification of group III sample 3)
- g3-3(40x) ex..tif (Microscope image, 40x magnification of group III sample 3 of extraction side)
- g3-3(40x) non ex..tif (Microscope image, 40x magnification of group III sample 3 of non- extraction side)
- g3-4 (10x).tif (Microscope image, 10x magnification of group III sample 4)
- g3-4(40x) ex..tif (Microscope image, 40x magnification of group III sample 4 of extraction side)
- g3-4(40x) non ex..tif (Microscope image, 40x magnification of group III sample 4 of non-extraction side)
- g3-5 (10x).tif (Microscope image, 10x magnification of group III sample 5)
- g3-5(40x) ex..tif (Microscope image, 40x magnification of group III sample 5 of extraction side)
- g3-5(40x) non ex..tif (Microscope image, 40x magnification of group III sample 5 of non-extraction side)
- g3-6 (10x).tif (Microscope image, 10x magnification of group III sample 6)
- g3-6(40x) ex..tif (Microscope image, 40x magnification of group III sample 6 of extraction side)
- g3-6(40x) non ex..tif (Microscope image, 40x magnification of group III sample 6 of non-extraction side)
- g3-7 (10x).tif (Microscope image, 10x magnification of group III sample 7)
- g3-7(40x) ex..tif (Microscope image, 40x magnification of group III sample 7 of extraction side)
- g3-7(40x) non ex..tif (Microscope image, 40x magnification of group III sample 7 of non-extraction side)
- g3-8 (10x).tif (Microscope image, 10x magnification of group III sample 8)
- g3-8(40x) ex..tif (Microscope image, 40x magnification of group III sample 8 of extraction side)
- g3-8(40x) non ex..tif (Microscope image, 40x magnification of group III sample 8 of non-extraction side)

Harvard Dataverse: Dataset4. Group IV samples. https://doi.org/10.7910/DVN/OLZF0L ^31^

This project contains the following underlying data:

- g4 (100x) ex.1.tif (Microscope image, 100x magnification of group IV sample of extraction side)
- g4 (100x) ex.2.tif (Microscope image, 100x magnification of group IV sample of extraction side)
- g4 (100x) non ex.1.tif (Microscope image, 100x magnification of group IV sample of non-extraction side)
- g4 (100x) non ex.2.tif (Microscope image, 100x magnification of group IV sample of non-extraction side)
- g4-1 (10x).tif (Microscope image, 10x magnification of group IV sample 1)
- g4-1(40x) ex..tif (Microscope image, 40x magnification of group IV sample 1 of extraction side)
- g4-1(40x) non ex..tif (Microscope image, 40x magnification of group I V sample 1 of non-extraction side)
- g4-2 (10x).tif (Microscope image, 10x magnification of group IV sample 2)
- g4-2(40x) ex..tif (Microscope image, 40x magnification of group IV sample 2 of extraction side)
- g4-2(40x) non ex..tif (Microscope image, 40x magnification of group IV sample 2 of non-extraction side)
- g4-3 (10x).tif (Microscope image, 10x magnification of group IV sample 3)
- g4-3(40x) ex..tif (Microscope image, 40x magnification of group IV sample 3 of extraction side)
- g4-3(40x) non ex..tif (Microscope image, 40x magnification of group IV sample 3 of non- extraction side)
- g4-4 (10x).tif (Microscope image, 10x magnification of group IV sample 4)
- g4-4(40x) ex..tif (Microscope image, 40x magnification of group IV sample 4 of extraction side)
- g4-4(40x) non ex..tif (Microscope image, 40x magnification of group IV sample 4 of non-extraction side)
- g4-5 (10x).tif (Microscope image, 10x magnification of group IV sample 5)
- g4-5(40x) ex..tif (Microscope image, 40x magnification of group IV sample 5 of extraction side)
- g4-5(40x) non ex..tif (Microscope image, 40x magnification of group IV sample 5 of non-extraction side)
- g4-6 (10x).tif (Microscope image, 10x magnification of group IV sample 6)
- g4-6(40x) ex..tif (Microscope image, 40x magnification of group IV sample 6 of extraction side)
- g4-6(40x) non ex..tif (Microscope image, 40x magnification of group IV sample 6 of non-extraction side)
- g4-7 (10x).tif (Microscope image, 10x magnification of group IV sample 7)
- g4-7(40x) ex..tif (Microscope image, 40x magnification of group IV sample 7 of extraction side)
- g4-7(40x) non ex..tif (Microscope image, 40x magnification of group IV sample 7 of non-extraction side)
- g4-8 (10x).tif (Microscope image, 10x magnification of group IV sample 8)
- g4-8(40x) ex..tif (Microscope image, 40x magnification of group IV sample 8 of extraction side)
- g4-8(40x) non ex..tif (Microscope image, 40x magnification of group IV sample 8 of non-extraction side)

Harvard Dataverse: main manuscript data. https://doi.org/10.7910/DVN/9IA7CS ^32^

This project contains the following underlying data:

- trough length.tab (Excel spreadsheet of measured trough lengths)
- taste buds number.tab (Excel spreadsheet of recorded taste bud numbers)

Data are available under the terms of the Creative Commons Zero "No rights reserved" data waiver (CC0 1.0 Public domain dedication).
