# Supplementary material for: Gustatory dysfunction in relation to circumvallate papilla’s taste buds structure upon unilateral maxillary molar extraction in Wistar rats: an in vivo study
Source: F1000Res. 2019 Sep 20;8:1667. [Version 1] doi: 10.12688/f1000research.19684.1 (PMC6947764; doi:10.12688/f1000research.19684.1)
Supplement: Peer review history

# Peer review: Reviewer response for version 1

**DOI:** 10.5256/f1000research.21589.r57946

**Published:** 2020-01-07

**Authors:** Mahmoud M. Bakr

**Affiliations:** 1 School of Dentistry and Oral Health, Griffith University, Gold Coast, Qld, Australia

**Abstract**:

 Some typos were evident, first line (teeth) should start with an upper case T as it is a new sentence. The same applies to (the) at the beginning of the results section.

 Also in the results section the authors report findings from groups II, III and IV without any previous definition of what each group consist of. The abstract should be readable as a separate entity without having to refer to the full article.

 In conclusions (which were more marked) should read (which was more marked) as it refers to the degenerative effect. Alternatively, the first sentence in the conclusions section should read "Maxillary molars extraction has degenerative effects....

**Introduction**:

 This section is well written but needs to be structured and reformatted into paragraphs rather than isolated sentences.

**Methods**:

 First paragraph, there is an extra bracket () before standard polycarbonate cages that needs to be deleted.

**Results**:

 Describing deformity of taste buds is very subjective and a more objective way of defining the degree of deformity is needed.

 Figure 4A is of a very poor quality and there are some concerns that it does not belong to this experiment at all due to different light conditions and degree/type of stain. It seems like a texbook image rather than an experimental one. Please replace this image.

 Unfortunately, nothing was reported about the differences between the four groups using a one-way ANOVA. Even if there were no significant findings, it should still be reported.

 For the independent t-test results, please report the actual P value and standard deviations rather than (P<0.05). This is important to inform the readers about the degree of variability within your samples and the level of statistical significance.

**Discussion**:

 It is recommended to discuss the difference that extraction of lower molars and wisdom teeth (in humans) would make to taste sensation as well.

**Conclusions**:

 It is misleading to report that extraction of maxillary molars had a degenerative effect on circumvallate papillae in general as no data from this study supports this statement. The data from this study only shows degeneration in comparison to the non-extraction side. Also the claim that the degeneration improves by time is only an observation and is not supported by statistical analysis (No data from the one way ANOVA) was presented.

 The conclusions need to rewritten both in the abstract and the full article text as it is over presenting the limited findings obtained from this study.

I confirm that I have read this submission and believe that I have an appropriate level of expertise to confirm that it is of an acceptable scientific standard.

# Peer review: Reviewer response for version 1

**DOI:** 10.5256/f1000research.21589.r54179

**Published:** 2019-10-07

**Authors:** Mahmoud M. Al-Ankily

**Affiliations:** 1 Oral Biology Department, Faculty of Dentistry, British University in Egypt, Cairo, Egypt

This article by Sana Mostafa *et al.* examines Gustatory dysfunction in relation to circumvallate papilla’s taste buds structure upon unilateral maxillary molar extraction in Wistar rats. The study, although it is simple, adds knowledge to the literature.

 Minor comments would help to improve the impact of this paper:

**Title:**

 Gustatory dysfunction in relation to circumvallate papilla’s taste buds structure upon unilateral **maxillary molar** extraction in Wistar rats but in **Methods** you said (all maxillary molars of the right side were extracted) so you may change it to **maxillary molars.**

**Introduction:**

 An interaction between dental and gustatory afferent neurons in the NST has been **previously discussed** please add a hint about it

**Methods:**

 In the experimental groups, all maxillary molars of the right side were extracted (early in the morning) why? Is the time of extraction make a significant difference.

 The tongue retracted by a tweezer. Postoperatively, antibiotics were administrated (cefotaxime10mg/Kg IV, Eipico, Egypt) for 3 days23. Analgesics were administrated (Ketoprofen (Anafen®, Merial) 5mg/kg I.M)24 once daily for up to 3 days did you do that with the control group? If not how to be sure that the medications does not affect the experiment.

**Results:**

 Figures from 2 to 6: please clarify which side is the extracted molars side and adjust the angulation of the CVP (figure 3 and 5) to make it easy to compare between the 2 sides of the trough.

**Discussion**

 Further investigations of the nerve supply under TBs of both sides using immunohistochemistry is recommended.

I confirm that I have read this submission and believe that I have an appropriate level of expertise to confirm that it is of an acceptable scientific standard.
